# Extracellular Vesicles in Aging: An Emerging Hallmark?

**DOI:** 10.3390/cells12040527

**Published:** 2023-02-06

**Authors:** Giorgia Manni, Sandra Buratta, Maria Teresa Pallotta, Davide Chiasserini, Alessandro Di Michele, Carla Emiliani, Stefano Giovagnoli, Luisa Pascucci, Rita Romani, Ilaria Bellezza, Lorena Urbanelli, Francesca Fallarino

**Affiliations:** 1 Department of Medicine and Surgery, University of Perugia, 06132 Perugia, Italy; 2 Department of Chemistry, Biology and Biotechnology, University of Perugia, 06123 Perugia, Italy; 3 Department of Physics and Geology, University of Perugia, 06123 Perugia, Italy; 4 Centro di Eccellenza sui Materiali Innovativi Nanostrutturati (CEMIN), University of Perugia, Via del Giochetto, 06123 Perugia, Italy; 5 Department of Pharmaceutical Sciences, University of Perugia, 06123 Perugia, Italy; 6 Department of Veterinary Medicine, University of Perugia, 06126 Perugia, Italy

**Keywords:** extracellular vesicles (EVs), senescence-associated secretory phenotype (SASP), senescence, aging

## Abstract

Extracellular vesicles (EVs) are membrane-enclosed particles secreted by cells and circulating in body fluids. Initially considered as a tool to dispose of unnecessary material, they are now considered an additional method to transmit cell signals. Aging is characterized by a progressive impairment of the physiological functions of tissues and organs. The causes of aging are complex and interconnected, but there is consensus that genomic instability, telomere erosion, epigenetic alteration, and defective proteostasis are primary hallmarks of the aging process. Recent studies have provided evidence that many of these primary stresses are associated with an increased release of EVs in cell models, able to spread senescence signals in the recipient cell. Additional investigations on the role of EVs during aging also demonstrated the great potential of EVs for the modulation of age-related phenotypes and for pro-rejuvenation therapies, potentially beneficial for many diseases associated with aging. Here we reviewed the current literature on EV secretion in senescent cell models and in old vs. young individual body fluids, as well as recent studies addressing the potential of EVs from different sources as an anti-aging tool. Although this is a recent field, the robust consensus on the altered EV release in aging suggests that altered EV secretion could be considered an emerging hallmark of aging.

## 1. Introduction

The term extracellular vesicles (EVs) defines membrane-delimited particles secreted by almost all types of cells. In the last decades, an increasing number of researchers have been interested in EVs as these nano/microsized vesicles mediate cell-to-cell communication in several physiological (i.e., immune response, tissue repair) and pathological (i.e. cancer, neurodegeneration, inflammation) processes [1]. The molecular cargo of EVs mirrors the physio-pathological state of the releasing cells [2,3]. This feature, together with the possibility to isolate EVs from biofluids, the ability to cross biological barriers (i.e., blood–brain barrier, blood–testis barrier, blood–retina barrier), and the stability in the gastro-intestinal tract have raised interest in EVs as innovative diagnostic and therapeutic tools.

Cells release different types of EVs that might be classified based on their biogenesis, density, size, internal cargo, and surface molecules. According to their origin, EVs are classified into exosomes, microvesicles (MVs), and apoptotic bodies. Exosomes, which originate from the inward budding of late endosomes, are first collected into multivesicular bodies (MVBs) and then secreted upon fusion with the plasma membrane [4]. MVs are formed directly by the outward budding of the plasma membrane, while apoptotic bodies are released by cells undergoing apoptosis [4]. Every single cell might release different EV classes and the current isolation methods produce EV preparations enriched in a specific population but containing other EV subtypes as contaminants. The heterogeneity of EVs released by a single cell introduces a level of complexity where it is necessary to understand the role played by EVs in a specific biological process. In this case, it would be necessary to isolate a “pure EV population” which should be devoid of contaminants, such as soluble molecules or other EV classes.

The correct nomenclature of the EV subtypes is an important issue to consider, as demonstrated by the fact that the biogenesis-based term exosome is very often used in an improper manner in the literature. In fact, this term has been often used for vesicles whose diameter was <100–200 nm without evidence regarding their endosomal origin [5]. The Minimal Information for Studies of Extracellular Vesicles (MISEV) guidelines propose several criteria for EV classification based on size (small EVs < 100 or <200 nm and medium/large EVs > 200nm) and density (light, medium, or heavy), or based on EV biochemical composition [5]. Notably, the MISEV guidelines recommend the use of the term “Extracellular Vesicle” when the characterization steps do not provide certain information regarding their intracellular origin [5].

The purity of EVs isolated by different methods can be determined by adequate biophysical and biochemical characterization steps. The biochemical cargo of EVs, which includes proteins, nucleic acids, and lipids, is the result of highly controlled but still poorly understood mechanisms of cellular selection, that discriminate those molecules that have to be included in EVs from the ones that have to be excluded. Conceivably, physio-pathological conditions and specific stimuli modulate certain biogenesis pathways and induce the release of different EV subtypes carrying unique molecular cargo. 

Regarding protein cargo, studies demonstrate that highly purified exosomes contain both conserved and cell-type-specific proteins, whereas proteins deriving from intracellular compartments (i.e., endoplasmic reticulum) are absent [6]. The set of conserved proteins includes those involved in MVB biogenesis (Alix, Tsg101), MVB transport and fusion with plasma membranes (Rab GTPase, annexins, flotillins), cell metabolism (glyceraldehyde-3-phosphate dehydrogenase and enolase-1) or proteins mainly associated to membrane microdomains, like integrins and tetraspanins (CD63, CD9, CD81, and CD82) [7,8]. Based on this evidence, Alix, Tsg101, CD63, and CD9 are considered typical exosomal markers. Nevertheless, it is now evident that, even if these proteins are enriched in exosomes, they are also present in other vesicle types [9]. In fact, among proteins associated with MVs (i.e., integrin, selectins, CD40 ligands, CD63, CD81, CD9) [10] are several exosomal markers. A recent study indicates annexin A1 as an exclusive marker for MVs [11].

Kowal et al. [9] classify vesicles isolated by differential centrifugation from media of human primary monocyte-derived dendritic cells in large EVs, pelleted at low speeds, medium EVs, pelleted at intermediate speeds, and small EVs, pelleted at high speeds. This study reveals also that different EV classes are present in the small EV fraction: small EVs co-enriched in CD63, CD9, CD81, and endosomal markers (i.e., bona fide exosomes); small EVs lacking CD63 and CD81 but enriched in CD9 and associated with endocytic and plasma membrane markers; small EVs lacking CD63, CD9, and CD81; and small EVs enriched in extracellular matrix proteins or serum-derived factors in the absence of endosomal signatures [9]. Notably, two recent studies demonstrate the presence of non-vesicular components, called extracellular nanoparticles [11], and small lipidic structures, called exomeres [12], within the bulk of small EVs. Altogether these data confirm that small EVs, which are generally defined as exosomes, might contain vesicular and non-vesicular structures derived from different subcellular compartments.

An aspect that has engaged the attention of researchers is that EVs contain nucleic acids that are transferred to target cells. The role of EVs in the horizontal transfer of genetic material became clear after the discovery that vesicular mRNAs can be translated into target cells [13]. More recently, it has been demonstrated that EVs carry a plethora of RNAs (i.e., miRNA, small interfering RNA, vault RNA, transfer RNA, long non-coding RNA, Y RNA, piwi-interacting RNA, and small nucleolar RNA), which might play functional roles in recipient cells [14,15]. EVs also contain DNA molecules, such as mitochondrial DNA [16], single-stranded DNA [17], and double-stranded DNA fragments [18]. 

As for proteins and nucleic acids, cells select the set of lipids forming EV membranes [19]. As a common feature, EVs are enriched in cholesterol, sphingolipids, ceramide, and glycerophospholipids containing saturated fatty acids [20,21,22]. This lipid asset, which resembles that of lipid rafts, determines the EV membrane stiffness [23,24] and stability in biological fluids [25]. EVs contain not only lipids forming the bulk of their delimiting membranes, but also carry lipid-derived mediators (i.e., prostaglandins, leukotrienes, pro-resolvins, and lysophospholipids) that play important roles in cell signaling. EVs can autonomously synthesize these lipid mediators since they contain phospholipases, which act on EV membrane phospholipids releasing the fatty acids that, in turn, can be converted by EV enzymes into bioactive molecules [26,27,28]. Notably, EVs participate also in the transcellular synthesis of leukotrienes and prostaglandins, by exchanging intermediates and enzymes involved in their synthesis between immune cells [27]. Altogether these results indicate that EVs possess a distinctive and ubiquitous molecular cargo. It is also worth mentioning that EV molecular signature also reflects the releasing cell type and its pathological status. For this reason, body fluid EVs have been recognized as a new powerful diagnostic tool.

The uptake of EVs by recipient cells is another important issue that has to be considered in vesicle-mediated cell-to-cell communication. EVs transfer signals into recipient cells through several mechanisms (i.e., activation of surface receptors, internalization by endocytosis, or direct fusion with the plasma membrane), depending on EV origin and recipient cell identity [10]. The ability of EVs to induce functional responses and phenotypic changes in target cells determines their potential use as therapeutic tools for clinical application. 

Several extra and intracellular stimuli can affect EV release. Among them, aging and cell senescence have been demonstrated to be characterized by changes in the amount and composition of released EVs. This evidence is so compelling that it can be proposed as an additional hallmark of aging. In this review, we are going to summarize studies investigating the roles of EVs in cell senescence and aging. We also report investigations showing the possibility to use EVs for therapeutic applications in the aging field. Since these data have been obtained by functional and biochemical analyses of EVs isolated from various sources (cell culture media or biofluids), using different isolation methods, it is difficult to assign a specific role in these phenomena to a unique EV subtype. For this reason, we have decided to use the term “Extracellular Vesicles" throughout the review, although in reported literature different definitions were used.

## 2. Aging 

Aging is defined as the progressive deterioration of an organism, resulting in the progressive impairment of tissue and organ physiological functions [29]. It is characterized by a chronic, sterile, and low-grade inflammation which contributes to the development of age-related diseases, including neurodegeneration, metabolic syndrome, and cardiovascular disorders [30]. The reduction in the ability to manage stress, together with the progressive increase in inflammation that characterizes the aging process has been defined as inflammaging [31]. In this context, a new field of research emerged, called geroscience. It has been developed on the assumption that mechanisms driving aging overlap with those driving age-related diseases, and it tries to unveil the molecular relationship between aging and age-related chronic diseases [32]. To this aim, the trans-NIH Geroscience Interest Group proposed the seven pillars of aging which include stress, macromolecular damage, metabolism, proteostasis, epigenetics, stem cells and regeneration, and inflammation [33]. It is important to underline that such pillars are strongly interconnected and affect each other, thus representing an intricated network that influences aging and age-related diseases. 

The seven pillars of aging partially overlap with the nine hallmarks of aging proposed by Lopez-Otin and colleagues in their seminal paper in 2013 [34], to define the causes of age-dependent accumulation in cellular damage [34]. The hallmarks of aging have been subdivided into three main categories: primary, antagonistic, and integrative, which act hierarchically. The primary hallmarks, i.e., nuclear, and mitochondrial genomic instability, telomere erosion, epigenetic alteration, and defective proteostasis, are characterized by their unequivocal participation in the aging process. In fact, any primary hallmark negatively affects cell behavior and leads to aging. On the other hand, antagonistic hallmarks have opposite effects based on their intensity and duration. Antagonistic hallmarks include mitochondrial dysfunction, associated with reactive oxygen species (ROS) generation, deregulated nutrient sensing, and cellular senescence. They initially aim at protecting the cell or the organism from injuries caused by the primary hallmarks but, once chronic, they worsen the damage. Integrative hallmarks, comprising stem cell exhaustion and intercellular communication, directly affect tissue/organism homeostasis. Thus, the antagonistic hallmarks tipping the balance for successful aging should be considered to prevent the insurgence of age-related chronic diseases, as discussed below.

The panel of the research symposium “New Hallmarks of Aging”, held in Copenhagen (Denmark) on the 22nd of March 2022, besides adding new hallmarks, as discussed below, acknowledged that the hallmarks of aging have greater value when viewed as a network rather than individual processes, and that attention to the interconnectedness of different hallmarks should be paid [35]. 

### 2.1. Mitochondrial Dysfunction Associated with ROS Generation

All the primary hallmarks of aging are linked to alteration in the genetic material and its principal metabolic products, proteins, in agreement with the finding that several progeroid syndromes are characterized by DNA damage accumulation [36,37,38]. 

DNA damage can be induced by endogenous threats such as DNA replication errors and ROS generation [39,40]; both events increase with age and contribute to the insurgence of age-related diseases [41]. DNA damage may also arise from environmental clues, although their specific influences on aging, apart from obvious causes such as smoking and obesity, remain poorly understood [33]. When DNA damage impacts stem cells and compromises their self-renewal properties (an integrative hallmark of aging), tissue functions might be lost [42]. Not only nuclear, but also mitochondrial DNA (mtDNA) is a target for damaging events contributing to the aging process [43]. mtDNA damage can affect mitochondrial fitness by dampening electron transport chain efficiency which, in turn, causes a decrease in ATP production, an accumulation of oxidized NAD^+^, and an increase in ROS generation. The observed increase in ROS levels with age led to the “free radical theory” of aging proposed by Harman in 1956 [44]. Moreover, ROS also plays a fundamental role in cellular signaling, specifically redox signaling, regulating the functioning of every cell in the organism. Therefore, Sohal and Orr proposed the “redox stress hypothesis” of aging [39], stating that the aging-associated functional decline is primarily driven by the disturbance in redox signaling, i.e., by the imbalance in either oxidative or reductive stress [45]. The concept of redox signaling is one of the reasons to consider mitochondrial dysfunction as an antagonistic hallmark of aging. Low-grade mitochondrial dysfunction, indeed, exerts positive effects on the aging process by lowering the ability to synthesize ATP through oxidative phosphorylation which, in turn, leads to the slowing of the aging process. In fact, as further discussed below, a calorie-restricted diet and intermittent fasting exert beneficial effects on prolonging life span by decreasing metabolic rate and ROS production [46,47]. 

On the other hand, an increase in ROS production can induce mtDNA damage which has been connected to the activation of the inflammatory responses. In particular, since mitochondrial DNA resembles bacterial genomes, it can be recognized by, and activate, different receptors linked to the immune response, including endosomal TLR9 (toll-like receptor 9) and cytoplasmic inflammasome receptors, which contribute to the onset of inflammatory responses [48]. As an example, mtDNA released by damaged mitochondria activates intracellular inflammasome receptors NLRP3 (NLR family pyrin domain containing 3) and AIM2 (absent in melanoma 2), thus leading to the secretion of the pro-inflammatory cytokine IL-1β and IL-18 [49,50] (see below for further discussion). 

In addition to mtDNA damage, an increase in ROS level may induce defective mitophagy, thus dampening the selective removal of malfunctioning mitochondria [43,51]. The ubiquitin-proteasome pathway components E3-ubiquitin ligase Parkin and PTEN-inducible putative kinase 1 (PINK1) are involved in the mitophagic process and recognized as Parkinson's disease (PD)-linked enzymes [52,53]. 

The increase in ROS generation caused by mitochondrial dysfunction might also affect proteostasis, a primary hallmark of aging [54,55]. The pathogenesis of several age-related diseases has been linked to protein aggregation possibly due to impaired proteostasis, such as the formation of β-amyloid fibrils and neurofibrillary tangles in Alzheimer’s disease, α-synuclein aggregates (Lewy body) in PD, huntingtin aggregates in Huntington’s disease, and islet amyloid polypeptide aggregates in type 2 diabetes [51,56,57,58,59,60,61,62,63]. This could explain why, when mitochondrial dysfunction becomes chronic, it can further increase the rate of the aging process by impairing proteostasis.

The alteration in cellular proteostasis may depend on several factors, including accumulation of aggregated proteins due to DNA mutation or incorrect protein folding, as well as deficits of protein clearance pathways such as ubiquitin-proteasome and autophagy-lysosome pathways, the latter now recognized as a self-standing hallmark of aging [35]. Since these processes are strictly dependent on intracellular ATP levels, it can be postulated that mitochondrial and protein quality control act synergistically to influence age-associated phenotypes. 

Moreover, disturbed proteostasis in synergy with inflammation boosts the aging process as demonstrated by the discovery that pro-inflammatory cytokines cause the replacement of the canonical catalytic β subunits in the proteasome complex, leading to the assembly of the immunoproteasome, which selectively degrades proteins involved in inflammation and immune response [64,65]. On the other hand, the inhibition of the immunoproteasome prevents the expression of pro-inflammatory cytokines [66]. Furthermore, increased expression of the immunoproteasome has been demonstrated in AD and amyotrophic lateral sclerosis (ALS) patients, and single nucleotide polymorphisms in immunoproteasome subunits increase the risk of developing neurodegenerative diseases [67]. Thus, mitochondrial dysfunction and the associated ROS generation are deeply interconnected with the other hallmarks of aging and strongly contribute to the fate of an aging cell/organism. 

### 2.2. Deregulated Nutrient Sensing 

As previously mentioned, a calorie-restricted diet and intermittent fasting decrease metabolic rate and ROS production, exerting beneficial effects on the aging process [46,47]. This discovery unequivocally demonstrated that the aging process is strictly dependent on nutrient availability and nutrient-sensing pathways.

Nutrient sensing through insulin/insulin-like growth factor (IGF) was the first pathway proved to regulate the aging process and to be involved in the insurgence of age-related diseases [68,69]. The insulin/IGF-1 signaling has been involved in the aging process by finding that organisms with a constitutively decreased signaling have a longer lifespan, probably because of the decrease in metabolic rate which, as previously discussed, might induce less cellular damage [70]. The insulin/IGF-1 signaling, via the insulin receptor substrate (IRS) adapter, activates phosphoinositide 3-kinase (PI3-K), which, in turn, triggers Akt and mTOR-1 activation. The insulin/IGF1 pathway can be considered an antagonistic hallmark of aging, since reduced mTOR signaling has been associated with longevity, whereas the increased activation of mTOR signaling characterizes the aged phenotype [71]. It is notable that mTOR inhibition lessened mitochondrial dysfunction [72] and protects from genomic instability and telomere attrition in preclinical model organisms [73]. 

Besides mTOR, AMPK (AMP-activated protein kinase) and sirtuins have also been involved in the aging process through the regulation of nutrient sensing. The decrease in intracellular energy availability can indeed be sensed by both AMPK, which responds to high AMP levels, and sirtuins, histone deacethylases responding to high NAD^+^ levels. AMPK, for example, can phosphorylate mTOR at Thr2446 leading to its inhibition [74] and thus protecting from age-related diseases. On the other hand, pharmacological, genetic, or stimulus-induced ablation of/reduction in sirtuin activity, leads to premature aging [75,76]. These findings agree with the observed decrease in NAD^+^ cellular levels during aging [77]. Moreover, nutrient availability controls the function of nicotinamide phosphoribosyltransferase (NAMPT), the rate-limiting enzyme of the NAD^+^ salvage pathway, with calorie restriction increasing its expression [78]. 

It must also be underlined that sirtuin activity defects, by affecting epigenetic modifications, alter cell epigenetic signature which can be inherited transgenerationally, thus potentially impacting the cell progeny. This is the case for the “trained memory” of innate immune cells which is mediated by epigenetic modification of immune cells (both monocytes and microglia) and has been suggested to produce a long-term hyperinflammatory status sustaining the aging process [76,79]. 

Besides genome integrity and epigenetic regulation, during the aging process, RNA processing changes also occur. The finding that interventions reversing the senescent phenotype seem to act by restoring splicing factor expression [80] suggested including the dysregulation of RNA processing between the new hallmarks of aging [35]. 

Furthermore, the aging process also provokes notable changes in the gut microbiome [81], including shifts in microbial populations and loss of species diversity. Together with the age-associated loss of structural integrity of the gut and of the blood–brain barrier these changes in microbial populations can drive inflammation. These observations led to the inclusion of microbiome dysfunction among the new hallmarks of aging [35]. 

### 2.3. Cell Senescence

Primary hallmarks of aging can induce cell senescence, a complex and multifaced process that results in an irreversible proliferation arrest, and resistance to apoptosis. Although senescent cells remain viable, they undergo alterations in metabolic activity [82]. An increase in senescent cell number during the aging process has been reported for several tissues and can reflect both an increase in the rate of senescence and a decrease in senescent cell clearance [34,79,82]. Cell senescence has been acknowledged as an antagonistic hallmark of aging, since it can be a compensatory response to protect the tissue from the proliferation of damaged cells, but if the senescent cell replacement process fails, the accumulation of senescent cells may worsen the damage, thus further contributing to the aging process. 

When the senescence program hits stem cells in a tissue, it can cause stem cell exhaustion (integrative hallmark of aging), thus leading to a decline in the regenerative potential culminating in tissue/organ dysfunction [32,34,83]. On the other hand, when the senescence program hits innate and adaptive immune cells, it induces inflammaging, the chronic and sterile inflammation distinctive of the aging process [79,84]. The senescence of immune cells, specifically termed immunosenescence, has been used to construct the inflammatory clock of aging (iAge), a deep-learning method based on patterns of systemic age-related inflammation and immunosenescence. iAge predicts important aging phenotypes and can be utilized for the early detection of age-related clinical manifestations [85].

A key characteristic of senescent cells is the secretion of a specific pattern of molecules collectively known as senescence-associated secretory phenotype (SASP). SASP includes a plethora of soluble signaling factors, such as pro-inflammatory cytokines, chemokines, angiogenic factors, bioactive lipids, and matrix metalloproteinases (MMPs) [86,87,88], which affect the tissue microenvironment potentially driving a self-propelling senescent phenotype. 

It has been shown that SASP depends on NAMPT expression and that endothelial SASP impairs insulin signaling in adipocytes [89,90]. These findings corroborate the bidirectional association between cell senescence, dysregulated nutrient sensing, and mitochondrial dysfunction. The pro-inflammatory nature of SASP, the involvement of chronic inflammation in fuelling the self-propelling damaging loop culminating in the insurgence of age-related chronic diseases, and its interconnections with other hallmarks, lead to the recognition of inflammation itself as a new hallmark of aging [35].

One of the pro-inflammatory molecules in SASP is IL-1β, whose secretion requires the inflammasome, a molecular platform that, in response to PAMPs (pathogen-associated molecular patterns) or DAMPs (damage-associated molecular patterns), gets activated and mediates pro-caspase-1 activation via auto-catalytic proteolysis. Active caspase-1, in turn, processes IL-1β that, in its mature form, can be secreted [91,92]. A pivotal role of the inflammasome in aging is demonstrated by the finding that mice lacking the NLRP3 inflammasome receptor have a longer health span and the use of small molecule NLRP3 inhibitors prevents age-related diseases in mice [93]. Moreover, an increase in IL-1β levels in biological fluids has been detected in patients affected by different age-related diseases [94,95,96,97], and inflammasome activation strongly contributes to both inflammaging and immunosenescence [98,99]. 

**Figure 1:**
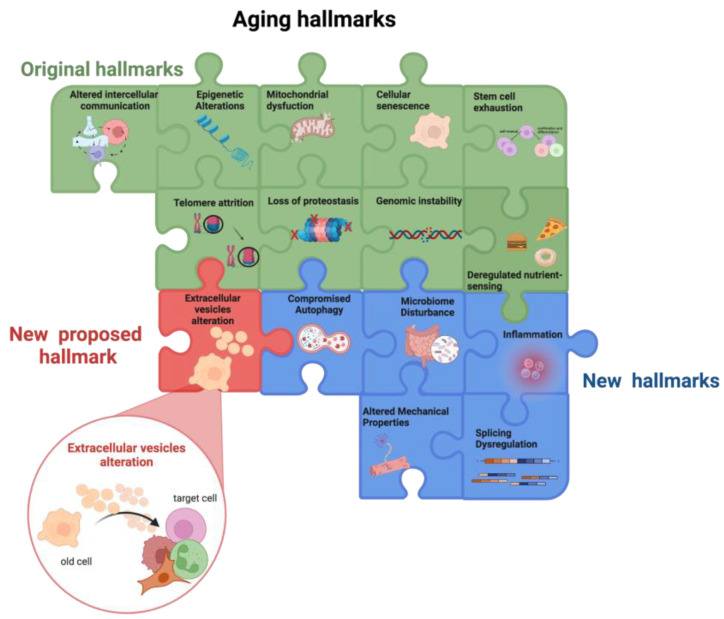
Aging hallmarks. The colored puzzle pieces represent the different hallmarks of aging: in green the original hallmarks and in blue the new hallmarks defined at the Copenhagen Meeting on Aging 2022. In this review, we propose “*the alteration in extracellular vesicles*” as an additional new hallmark of aging (in red). Other potential new hallmarks emerging from future studies will lead to puzzle completion. Created by BioRender.com (accessed on 18 January 2023).

SASP contains not only soluble factors but also EVs [82]. These, besides being involved in intercellular communication, have recently gained attention in the context of aging, as their release is altered by aging and, in turn, they strongly impact the aging process, as discussed in Section 3 (Figure 1).

Overall, the main factors involved in the aging process, i.e., the nine hallmarks of aging, influence each other thus creating a vicious cycle that promotes the aging process. Moreover, the bidirectional association between the hallmarks of aging and inflammaging fuels a self-propelling damaging loop culminates in the insurgence of age-related chronic diseases (Figure 1).

## 3. EVs in Aging and Senescence 

### 3.1. Cellular Senescence and EVs

Many studies have investigated the release of EVs by cells undergoing senescence and there is a quite robust consensus that cell senescence is associated with an increase in EV release. Moreover, senescent EVs show different biochemical content and can spread senescence signals in the neighboring cell/tissue. Cultured cells can be induced into senescence by a variety of stimuli. In several studies, cell senescence was induced by serial replication: cells at early passages were considered young, whereas cells subjected to multiple passages of proliferation were considered old. It has been demonstrated that, compared to young human dermal fibroblasts (HDFs), senescent HDFs produce relatively higher levels of EVs, which hampers keratinocyte differentiation and increases the level of proinflammatory cytokine IL-6 [100].

Similarly, Riquelme et al [101] induced replication until human umbilical vascular endothelial cells (HUVECs) reached senescence and observed that senescent cells produced an increased number of small EVs. Mensà et al [102] also reported that senescent HUVEC cells released a greater number of small EVs, enriched in miR-21-5p and miR-217. These miRNAs induced an impairment of DNA methyltransferase 1 and Sirtuin 1 expression, leading to epigenetic changes and to the acquisition of a senescent phenotype in recipient cells.

Other studies investigated senescence induced by DNA damage or oncogene activation. In a pioneering study on DNA damage induced by irradiation, Lehmann et al. [103] observed that clinically relevant doses of radiation induced premature senescence in human prostate cancer cells, and that this state was associated with a significant increase in EVs in the extracellular environment. Interestingly, in this first work, the authors also reported that the release of these vesicles was dependent on p53 activation. Later, other studies confirmed the relevance of p53 for EV release. For instance, a proteomic approach employed to identify proteins secreted after DNA damage (a primary hallmark of aging) demonstrated that cells release EVs in a p53-dependent manner [104], and when TSAP6 (tumor suppressor-activated pathway 6) expression, which is a direct p53 transcriptional target gene, was abrogated, EV production was severely compromised [105]. More recently, Tesei et al [106] also reported a pivotal role of p53 in regulating EV release upon its activation by high radiation doses. EVs released upon irradiation decreased telomerase activity in recipient cells [107], possibly because telomeric repeat-containing RNA (TERRA), which is known to repress telomerase activity [108], is enriched in EVs [109,110]. It is important to highlight that telomere erosion is considered a primary hallmark of aging. In HDFs, senescence induced by oncogene activation is associated with an increased release of small EVs, with a peculiar fatty acid and lipid signature [111,112]. A possible molecular mechanism responsible for the increased EV release in senescent cells has been investigated by Hitomi et al. [113]. This work revealed the importance of the ceramide synthetic pathway for EV release, as in senescence induced by doxorubicin treatment the higher EV release was correlated with a downregulation of sphingomyelin synthase 2 (SMS2) and an upregulation of neutral sphingomyelinase 2 (nSMase2). 

A few studies have focused their attention on the induction of proliferation and invasion in target cells, mediated by EVs derived from senescent cells. Fibroblasts undergoing (i) replicative senescence, (ii) transforming growth factor β1-induced senescence, or (iii) isolated from human subjects with an age-related lung disorder, released a higher number of EVs inducing an invasive phenotype in recipient cells, which was dependent on fibronectin localized on the EV surface [114]. Al Suraih et al. [115] observed that EVs from senescent cholangiocytes promote the proliferation of normal cholangiocytes, while Takasugi et al. [116] showed that small EV-associated EphA2 secreted from senescent cells binds to ephrin-A1, promoting cell proliferation through EphA2/ephrin-A1 reverse signaling. HDFs induced to senescence by H_2_O_2_ treatment showed a four-fold increase in the release of small EVs containing miRNAs predicted to target mRNAs of pro-apoptotic proteins [117]. On the other hand, EV release may also be involved in maintaining homeostasis in the releasing cell. In fact, inhibition of EV secretion induced the accumulation of nuclear DNA in the cytoplasm, provoked the innate immune response, and caused the activation of cytoplasmic DNA sensing machinery, in turn leading to ROS-dependent DNA damage response and to the induction of a senescence-like cell-cycle arrest or apoptosis in normal human cells [118].

In addition to DNA-damage-induced genome instability, mitochondrial dysfunction, another primary hallmark of aging preceding cell senescence, has been also reported to affect EV release. A recent study observed that mitochondrial components, including mtDNA, are present in EVs [119], suggesting that EVs could transfer mtDNA in target cells, eliciting functional changes, such as innate immune response. In agreement with this finding, Wang et al. [120] reported that the increased mtDNA damage associated with normal aging alters the functionality of the retinal pigment epithelium (RPE) in age-related macular degeneration, with a molecular mechanism involving EV release of intracellular proteins.

Stem cell exhaustion is considered an integrative hallmark of aging because the loss of stem cell functionality is strongly connected to dysfunction of tissue homeostasis. Mesenchymal stem cells (MSCs) induced to senescence by multiple passages of replication have been investigated since these cells are generally regarded as a source of EVs for regenerative purposes in cell-free therapies. Therefore, the study of molecular and morphological changes associated with multiple passages of cultivation is of key importance for translational approaches. Age-related alterations in EV release by MSCs have been associated with their dysregulated function [121] and Lei et al. [122] found that senescent, late-passage MSCs secrete higher levels of EVs with smaller sizes and a decreased CD105+ level. 

### 3.2. Body Fluid EVs and Aging 

Several studies have analyzed body fluid EVs from different aging models (mice, rats, humans), aimed at elucidating the features of body fluid EVs (number and size distribution) and their different biochemical content or biological properties, between old and young subjects. 

In terms of amount, there is no consensus whether the EV number in different body fluids is increased in old vs. young subjects. A couple of studies have reported that EV concentration decreases with age in human plasma (30 to 60 years old) [123] and rat serum (3 vs. 21–23 months old) [124]. On the other hand, other studies have observed that a larger number of EVs are present in bodily fluids from older subjects compared to younger subjects, namely in human plasma (20 to 30 vs. 80 years old) [125,126], mice plasma (3 vs. 18-21 months old) [127], human follicular fluid (<35 vs. >38 years old women) [128]. A different result was obtained by Alberro et al. [129], which described the concentration of human serum EVs as not affected by age when individuals were grouped into adults (20–49 years old) and elders (70–104 years old). As for size, Zhang et al. [124] compared the size distribution of EVs from the serum of young and old rats, reporting that old EVs have a larger average diameter, whereas Alibhai et al. revealed that old EVs from mice plasma had a smaller size [127]. These studies were carried out on different models, using different sources of blood EVs (serum, plasma), and different separation methods (polymer precipitation, centrifugation), and focused on different EV subtypes based on size, so they are difficult to compare. Moreover, the EVs size distribution in young vs. old subjects’ body fluid EVs was scarcely investigated. Further studies based on internationally recognized guidelines are needed to obtain reliable evidence on the variability of EV number and size distribution with age. For example, current MISEV guidelines do recommend the use of plasma and not of serum, as the activation of the clotting cascade may significantly alter the number of recovered EVs. 

The biochemical content investigation on body fluid EVs from young and old subjects evidenced that EVs are different in terms of proteins, lipids, and nucleic acids, namely miRNAs, and mtDNA. As for proteins, CD63 is considered one of the best available markers for EVs. Interestingly, Gomes de Andrade et al. [130] reported an age-related (3 vs. 21 months) decrease in CD63 level in rat plasma EVs and an increase in EVs from the cerebrospinal fluid (CSF). Similarly, an age-related linear decrease in mice and humans plasma EVs was observed for extracellular NAMPT (eNAMPT) [131], and for galectin-3 (<25 vs. >55 years old), which was found to protect β-catenin from degradation, ameliorating osteoblastogenesis [132]. Zhang et al. [133] observed that several specific plasma EVs from immune cells, carrying surface markers of B cells, T cells, NK cells, and APC, declined with aging (40 ± 18 years vs. 68 ± 8 years). Alberro et al. [129] measured T cell membrane markers on plasma EVs, showing that a higher percentage of CD28^-^ EVs was observed among CD8 EVs, which could be linked to the increase in CD28^-^ CD8 T cells with aging. On the other hand, a few studies described proteins whose level of EVs from circulation increased with aging. Elevated pro-BDNF (brain-derived neurotrophic factor) in total plasma EVs and in a subpopulation of plasma EVs enriched for neuronal origin (i.e., expressing the neuronal marker L1 cell adhesion molecule) was reported by Suire et al. [134]. Interestingly, the accumulation in EVs of proteins of pathological relevance with aging has also been reported. Koinuma et al. [135] observed that in aged cynomolgus monkeys, Aβ levels in EV/intraluminal membrane vesicles isolated from temporal lobe parenchyma were drastically increased, suggesting that abnormal accumulation of Aβ in EVs may be involved in age-dependent Aβ pathology. As for lipids, ceramide was observed to increase with aging in serum EVs isolated by size exclusion chromatography from young (24–40 years) vs. older (75–90 years) women and young (6–10 years) vs. older (25–30 years) rhesus macaques [136].

A large amount of work on body fluid EVs in aging has been dedicated to the comparison of miRNAs present in EVs from old and young subjects, looking for easily measurable aging biomarkers (Table 1). Specific miRNAs have been reported to be increased in body fluid EVs, namely miR-34a in mice serum (6 vs. 24 months) [137] and miR-31 in human plasma (<25 vs. >55 years) [138]. Alibhai et al. [127] screened miRNA expression in mice plasma EV fractions and identified nine miRNAs with a significantly different expression between young and old, as miR-146a, miR-21, miR-22, miR-223, miR-145, and let-7a were increased in old EVs and miR-212 and miR-455 in young EVs. miRNA profiling of small EVs from the plasma of healthy subjects aged 40–100 years showed an inverse U-shaped age-related trend for miR-21-5p [102]. In human follicular fluid from aged individuals, it was reported that CD81^+^ small EVs contained lower levels of miR-16-5p, miR214-3p, and miR-449a, and higher levels of miR-125b, miR-155-5p, and miR-372 [128]. In human salivary EVs miR-24-3p [139] was increased with aging. Comparative studies between old (24–28 months) and young mice (3–4 months) revealed that in EVs isolated from the bone marrow interstitial fluid, the miR-183 cluster (miR-96/-182/-183) was expressed at a higher level in aged EVs [140]. As for DNA, age-related changes in plasma mtDNA encapsulated in EVs were investigated by Lazo et al. [141], that observed that mtDNA levels decreased with age.

Several studies investigated the biological properties affected by EVs isolated from young and old subjects and there is consensus that EVs from old subjects are mediators of the progressive deterioration that eventually results in age-associated tissue dysfunction. Alique et al. [125] observed that plasma EVs from elderly human subjects, as well as those from senescent endothelial cells, were able to promote vascular calcification, whereas Qureshi et al. [142] reported that spleen leukocyte EVs from old rat spleen (3 vs. 16 months) were strong promoters of age-associated endothelial dysfunction. A reduced osteo-inductive potential was assessed for elderly subjects’ EVs [132], as well as a serum EVs contribution to cardiac fibrosis [143]. Oligodendrocyte differentiation supported by EVs released by astrocytes also decreased with aging [144].

**Table 1:** The table shows the main studies comparing miRNAs present in the EVs from biofluids of young and old subjects, listing the main species differentially expressed in aged vs. young subjects, potentially useful as aging biomarkers.

Organism	EVs Source	miRNA		Reference
		*Up Regulated*	*Downregulated*	
**Mice**	**Serum**	miR-34a		Fulzele et al., 2019 [136]
	Serum	miR-192		Tsukamoto et al., 2020 [145]
	Plasma	miR-146a miR-21 miR-22 miR-223 miR-145 let-7a	miR-212 miR-455	Alibhai et al., 2020 [127]
	Bone marrow interstitial fluid	miR-96 miR-182 miR-183		Davis et al., 2017 [139]
Human	Plasma	miR-31		Weilner et al., 2016 [137]
	Plasma	miR-21-5p		Mensà et al., 2020 [102]
	Follicular fluid	miR-125b miR-155-5p miR-372	miR-16-5p miR-214-3p miR-449a	Battaglia et al., 2020 [128]
	Saliva	miR-24-3p		Matchida et al., 2015 [138]

## 4. Anti-Aging Potential of EVs from Various Sources

As previously introduced, in the last decades it has been widely recognized that EVs secreted from senescent cells can transfer the aging phenotype to the recipient cells by regulating their gene expression or changing their energy metabolism [146]. To date, just some studies underline the properties of specific EVs in delaying aging processes. Here we reviewed recent in vitro and in vivo studies that address the EVs as a potential anti-aging tool.

### 4.1. In Vitro Studies

Several studies point out that EVs derived from human induced pluripotent stem cells (iPSCs) and MSCs alleviate the senescence of human cells in vitro.

Fafián-Labora et al. [147] showed that incubation of old MSCs with young MSC-derived EVs decreased aging markers and increased pluripotency markers. By contrast, the incubation of young MSCs with old MSC-derived EVs reversed the effects. Wang et al. [110] provided evidence that bone marrow (BM)-MSC-EVs from young and old rats (3 and 24 months old) influenced tumor growth factor-beta 1 (TGF-β1)-mediated epithelial-mesenchymal transition (EMT) of renal cells. In fact, the treatment with BM-MSC-EVs derived from older rats significantly reduced their growth rate and cell migration, reducing the inhibitory effect on TGF-β1-mediated EMT in a weaker manner as compared to BM-MSC-EVs derived from younger rats. These findings suggest that age-related changes in EVs may play a role in fibrosis linked to aging [110]. Another study showed that EVs produced from aged MSCs possessed activated AKT signaling and when transferred to murine hematopoietic stem cells (HSCs) caused aging. In contrast, intercellular transfer of EVs collected from young MSCs or from aged MSCs treated with AKT inhibitors rejuvenated aged HSCs and restored their functionality [148]. Specifically, the authors showed that the anti-aging effect of EVs from young MSCs is mediated by miR-17 and miR-34a transfer in recipient cells that controlled the reduction of autophagy-related mRNAs. 

Studies were also reported for EVs released by iPSCs. Liu et al. showed that human iPSCs produce great numbers of EVs under defined culture conditions [149]. These iPSC-EVs contain a high concentration of the intracellular antioxidant protein peroxiredoxins and when applied to senescent MSCs they reduce intracellular ROS levels and alleviate their aging phenotypes [149]. Furthermore, the authors demonstrated that iPSC-EVs attenuate progerin-induced senescence in the premature aging cell model. Similarly, human iPSC-EVs ameliorated the aging of skin fibroblasts in vitro by increasing the expression level of collagen type I [150].

Additional data on the antiaging effect of iPSC-released EVs come from studies using senescent organoids as a potential new model for better understanding aging progression. It is known that stem cells and iPSCs-derived exosomes have anti-senescence effects in several age-related diseases such as primary biliary cirrhosis (PBC) and primary sclerosing cholangitis (PSC) [151]. Interestingly, recent data used novel organoid culture technology (cholangioids) to investigate the anti-aging potential of iPSC-EVs. Senescence of cholangioids was obtained by persistent oxidative stress exposure that increased the expression of senescence-associated proteins and SASPs. Treatment with iPSC-EVs delayed the aging progress of senescent cholangioids by reducing the levels of senescent-associated proteins, particularly cell-cycle associated inhibitor proteins p21WAF1/Cip1 and p16INK4a, and SASP components. These results establish an in vitro model of age-related diseases, including PSC, and for the first time developed a novel therapeutic approach using EVs 3D in vitro model. 

### 4.2. In Vivo Studies 

The effect of EVs was also addressed in some preclinical in vivo mouse models of senescence. Recent data from J. A. Fafian-Labora and colleagues [146] demonstrate the potential of small EVs isolated from primary fibroblasts of young human donors (sEV-Ys) as regenerative therapy in aging. Specifically, they reported that sEV-Ys possess glutathione-S-transferase (GST)-intrinsic activity and ameliorated cellular biomarkers of senescence by increasing the antioxidant capacity of old fibroblasts both in vitro and in vivo in old mice [146]. Indeed, the treatment of old mice (22–24 months of age) with sEV-Ys, administered intraperitoneally twice a week for three weeks, significantly reduced several biomarkers of senescence including lipid peroxidation. This important evidence highlights the potential of EVs as anti-aging therapeutic agents.

Circulating levels of eNAMPT significantly declined with age in mice and humans, as expected from its involvement in the deregulation of nutrient sensing (see paragraph 2.2). EVs containing eNAMPT were recently demonstrated to extend the health span and increase the physical activity performance of elderly mice [131]. This effect was dependent on the increased synthesis of NAD^+^ in various tissues, thereby enhancing tissue functions and extending lifespans in mice. To test the ability of eNAMPT-EVs to extend the lifespan of aged mice, the researchers isolated EVs from young (4-6-month-old-mice) mouse plasma or exogenously supplemented EVs with eNAMPT and injected them intraperitoneally into 20-month-old mice for four consecutive days. eNAMPT-EVs were able to mitigate age-associated functional decline in specific target tissues, including the hypothalamus, hippocampus, pancreas, and retina, and delayed age-associated mortality rate extending health span and lifespan in mice. In addition, the treatment of mice with EVs significantly enhanced wheel-running activity, extended the lifespan, and mitigated age-associated factors [131]. Interestingly, eNAMPT is also contained in EVs purified from human blood (see paragraph 3.2). Thus, these results open a new opportunity to use EVs containing eNAMPT from young individuals as anti-aging biologics in humans. 

In addition to specific enzymes, distinct miRNA in EVs was shown to promote anti-aging effects. In 2020 Tsukamoto H et al., demonstrated that EVs containing miR-192 were able to alleviate hyperinflammatory states and immune dysfunction in the elderly. Specifically, the authors showed that the hyperinflammatory state of the aged mice caused the production of EVs enriched in miR-192 that, in turn, exhibits anti-inflammatory effects on immune cells including macrophages [145]. Notably, in an aged mouse model, characterized by prolonged inflammation in the lung induced by intranasal administration of inactive influenza whole virus particles (WVP), intravenous administration of miR-192-EVs alleviated the hyperinflammatory state of the aged mice, resulting also in improved vaccination efficacy in aged mice. Therefore, aged-miR-192-EVs may constitute a negative feedback loop alleviating aging-associated immune dysfunction [145].

Anti-inflammatory activities have been reported in EVs purified from young blood. Although in the last two decades it was demonstrated that soluble factors contained in young blood are able to rejuvenate aged systems, including skeletal muscle and the CNS [152,153], only recently it has become evident that EVs from young serum are among the most important actors involved in the attenuation of inflammaging and in delaying age-associated disorders. In a work by Wang et al., authors report that EVs isolated from the serum of young mice (2–3 vs. 18–20 months) were capable of rejuvenating aged T cells, contributing to the rejuvenation of aged thymic function. These findings suggest a great potential for EVs from the serum of young individuals as novel therapeutic approaches to reduce morbidity and mortality caused by age-related inflammatory diseases [154].

EVs from different types of stem cells have been investigated for their antiaging effects in vivo. In an acute lung injury model, only EVs from MSCs of a young subject (25 vs. 72 years) exhibited protective effects [155]. The hypothalamus plays an important role in organism aging and aging retardation. It was reported that lifespan extension could be achieved in middle-aged mice that were locally implanted with healthy hypothalamic stem/progenitor cells. These cells contributed to the release of specific miRNAs in the CSF, whose level declined during aging. Notably, treatment with EVs from healthy hypothalamic stem/progenitor cells also led to the slowing of aging, providing evidence that hypothalamic stem cells control the aging speed via EVs.

Aging is also a risk factor for cardiovascular diseases. It was recently demonstrated that the administration of umbilical mesenchymal stem-cell-derived exosomes (UMSC-EVs) could prevent aging-induced cardiac dysfunction induced by the injection of D-galactose (D-gal) in mice [156]. Moreover, the authors explored the potential mechanism by which UMSC-EVs exerted their protective effects and demonstrated that UMSC-EV administration increased the anti-aging marker TERT while decreasing the aging p21 protein as well as the inflammatory cytokine TNFα and the NF-κB component p-p65 in cardiac cells. 

UMSC-EVs were also enriched in the lncRNA MALAT1 (metastasis-associated lung adenocarcinoma transcript 1), which is associated with cell senescence and regulates cell cycle and inflammation [157,158]. The anti-aging effect of UMSC-EVs was blocked by a siRNA directed to MALAT1 (siMALAT1), suggesting that their effect was mediated by MALAT1 lncRNA in this aging model [156]. This finding supports the idea that EVs can be used to deliver lncRNA with clinical implications in delaying the aging process. These data were confirmed in 2021 by Qian Lei et al., demonstrating that EVs derived from neonatal umbilical cord mesenchymal stem cells (UC-EVs) have a strong potential in rejuvenating senescing adult bone marrow MSCs by increasing their self-renewal capacity and telomere length, i.e., by reverting two primary hallmarks of aging [159]. Mechanistically, the authors showed that UC-EVs were able to transfer to bone marrow recipient cells the mRNA transcripts of proliferating cell nuclear antigen (PCNA). In this study, UC-EVs were tested therapeutically in naturally senile mice. Intravenous injection of UC-EVs in old mice slowed bone degeneration while enhancing wound healing and angiogenesis. Moreover, other positive effects associated with UC-EVs administration were disclosed including decreased oxidative stress and inflammatory mediators in the plasma and decreased age-related deterioration of bone and kidney [159]. These characteristics make UMSC-EVs a good candidate as a potential therapeutic agent. Another study published in 2020 by Feng et al. showed that iPS-MSC-EVs were capable of mitigating aging-associated arterial stiffness and hypertension [160]. Old mice treated with iPS-MSC-EVs via tail vein once a week for a total of four weeks showed a significant attenuation of endothelial dysfunction, arterial stiffening, and hypertension. In addition, mechanistic experiments showed that iPS-MSC-EVs attenuated arterial aging by activating the SIRT1-AMPKα-eNOS pathway and inhibiting MMPs and elastase [160] strengthening the idea that counteracting the hallmarks of aging leads to the amelioration of age-related pathologies.

Recently, in addition to these studies using umbilical stem-cell-derived EVs, it was reported the positive effects of EVs released by gingiva-derived mesenchymal stem cells (GMSC-EVs) in alleviating age-related cell senescence. Results from this study demonstrated that systemic administration of GMSC-EVs for eight weeks in old mice robustly reduced oxidative stress attenuating the level of aging-associated markers such as p21, mTOR, IL-6, and TNF-α in skin and heart tissues [161]. These findings suggest that GMSC-EVs could be used as potential new agents to inhibit senescence-related cellular genes in specific tissues. 

**Figure 2:**
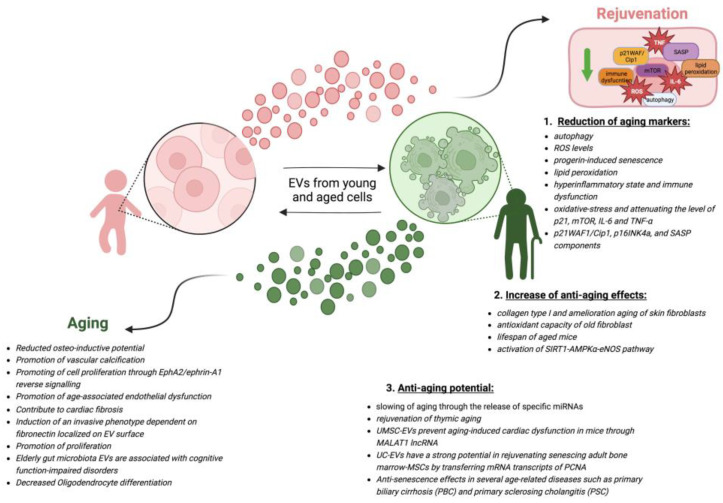
The role of EVs in aging. Extracellular vesicles are known to influence aging and age-related diseases. Both young and senescent cells are capable of secreting EVs. Secreted EVs from young and senescent cells can induce rejuvenation or aging in senescent and young recipient cells, respectively, as indicated by arrows. The molecular mechanisms by which EVs from young cells induce rejuvenation of old cells can be divided into 3 main groups: (**1**) reduction of aging markers [145,147,148,149,151,161], (**2**) increase of anti-aging effects [131,147,150,160], and (**3**) anti-aging potential [151,154,155,156,159]. The arrows between the two types of cells mean that both cells are able to secrete EVs. Relevant literature is provided for each group. Mechanisms by which EVs from senescent cells may promote aging and age-related diseases in young cells include promotion of vascular calcification, cardiac fibrosis, reduction of cell differentiation, and others. References to relevant publications on these topics are also provided [125,114,115,116,141,142,143,144,162]. Created with BioRender.com accessed on 20 January 2023.

Altogether these findings shed light on the therapeutic potential of EVs mostly derived from MSCs, for improving longevity (Figure 2). 

## 5. Microbiota-Derived EVs and Aging 

In the last few years, interest in microbiome EVs has increased in the medical field as biomarkers of metabolic diseases and cancers. The inclusion of microbiome dysfunction among the hallmarks of aging relies on the finding that the composition of the microbiome community (gut, feces, blood, and urine) is influenced by aging which, in turn, induces immune system dysregulation and susceptibility to age-related disorders. For this reason, microbiome EVs may be useful as biomarkers for alterations in the aging process. Furthermore, since alteration of the microbiota and microbiome EVs composition is dependent on several environmental factors, including age, it is possible to hypothesize microbial EVs as a new tool with therapeutic potential in anti-aging medicine.

A pilot study, published in 2020, showed for the first time the role of the gut microbiota EVs derived from elderly individuals in cognitive impairment [162]. The authors demonstrated that transplantation of EVs isolated from *Paenalcaligenes hominis* and *Escherichia coli*, enriched in the feces of elderly individuals, significantly caused cognitive impairment and colonic inflammation in recipient mice. This is the first evidence that EVs derived from the microbiota of old healthy subjects could be associated with impaired cognitive function, such as those present in Alzheimer’s disease. 

On the contrary, in 2021, the beneficial role of gut microbiota EVs from children in protecting against osteoporosis was reported [163]. In particular, authors identified in child gut microbiota the specific bacterial specie Akk (*Akkermansia muciniphila*), which is important for maintaining bone mass and strength, both lost in old subjects. Moreover, these authors also discovered a new communication mode between gut microbiota and bone through bacteria-derived EVs. 

Skin microbiome has also been the object of investigation. Chan Song Jo et al. demonstrated the ability of Lactobacillus plantarum EVs (LpEVs) derived from young women (20 years old) on suppressing skin aging factors. Firstly, the authors found that young women had a higher population of LpEVs in their skin microbiome compared to 50 years old women. In addition, LpEVs from young women induced cell proliferation, regulated extracellular-matrix degradation, and improved skin aging [164]. 

Overall, these recent findings underlie an interesting role of microbiota-derived EVs and suggest their potential role for anti-aging interventions.

## 6. Conclusions

Altogether, the data summarized in this review support the idea that alteration in EV secretion, (i.e., alteration in their levels, cargo, and diverse effects on target cells) could be considered a new hallmark of aging. Investigations on the role of EVs in aging show great potential for the modulation of age-related phenotypes and for pro-rejuvenation therapies beneficial for many diseases associated with aging, namely cancer, cardiovascular disease, diabetes, and neurodegenerative disorders. 

EV alteration with aging suggests that the modulation of EV release, in terms of number and content, could represent a target to slow aging and for the therapy of age-related diseases. First, the evaluation of EV features associated with aging (i.e., number, size, specific markers, genetic and/or biochemical content) could represent a possible biomarker of aging, useful to evaluate a variety of age-delaying therapeutic approaches. Second, the decrease of EVs number, via the removal of senescent cells which are known to release a higher number of EVs, could represent a rejuvenating tool associated with treatment with senolytic drugs. Third, the administration of EVs including key components showing anti-aging effects could be an effective rejuvenating strategy, potentially safer than the administration of whole cells. Nonetheless, these studies are still preliminary and key issues have still to be elucidated. These challenges require further studies assessing the specific content of aging-associated EVs, as well as the development of methods and tools to produce EVs containing rejuvenating factors in a safe and abundant manner. In cell models, there is agreement that cell senescence is associated with an increased release of EVs, but the functional role of these EVs is less clear, as a few studies have reported pro-senescent and pro-apoptotic effects, whereas others have described a pro-tumorigenic role. As for body fluid EVs, further studies are needed to assess whether aging is associated with an increased or decreased number of EVs, and the main biochemical features of EVs circulating in old vs. young individuals, both in humans and in animal models, need to be further elucidated. Indeed, it must be considered that these samples are subjected to mechanical, chemical, and thermal stress during sampling and processing. This may notably change the shape, size, and composition of EVs in a manner that could be possibly influenced by age-related factors.

However, there is converging evidence that EVs from young subjects have a rejuvenating effect and vice versa. Nevertheless, the isolation of pure EV subtypes from body fluids is still a debated issue, and it is mandatory to develop translational applications exploiting EVs for rejuvenating therapeutical approaches.
